# The Interaction Between Orthodontics and Periodontal Tissue Remodeling -

**DOI:** 10.31661/gmj.vi.4005

**Published:** 2025-09-22

**Authors:** Mina Abasi, Ali Goodarzi, Raheleh Solhmirzaei, Fatemeh Fazel, AmirMohammad Moharrami, Hamid Ghasemi, Haniyeh Alavi Milani

**Affiliations:** ^1^ School of Dentistry, Tehran University of Medical Sciences, Tehran, Iran; ^2^ Department of Periodontics, School of Dentistry, Shiraz University of Medical Sciences, Shiraz, Iran; ^3^ Department of Dentistry, Qazvin University of Medical sciences, Qazvin, Iran; ^4^ Faculty of Dentistry, Tehran University of Medical Sciences ,Tehran, Iran; ^5^ Department of Orthodontics, Faculty of Dentistry, Tehran University of Medical Sciences, Tehran, Iran; ^6^ Eline Orthodontic Clinic, 201, Negin Elahiye Tower, Shariati St, Tehran; ^7^ Pediatric Department, School of Dentistry, Tehran University of Medical Sciences, Tehran, Iran

**Keywords:** Orthodontics, Periodontal Remodeling, Tissue Engineering, Inflammation, Alveolar Bone

## Abstract

Orthodontic tooth movement (OTM) involves a complex cascade of biomechanical and biological events, orchestrated through the interaction of mechanical forces, inflammatory responses, and cellular remodeling within the periodontal ligament and alveolar bone. In adult patients, particularly those with a history of periodontitis, these processes are further complicated by reduced regenerative capacity, chronic inflammation, and altered bone dynamics. This review explores the mechanobiological foundations of OTM, detailing the roles of osteoclasts, osteoblasts, fibroblasts, and endothelial cells, as well as the central regulatory pathways, including RANKL/OPG signaling, cytokine cascades, and matrix metalloproteinase activity. Special attention is given to the clinical implications of orthodontic forces in healthy versus compromised periodontium, emphasizing the importance of force magnitude, direction, and regimen. Interdisciplinary coordination between orthodontists and periodontists is essential for safe and effective treatment planning, particularly when regenerative procedures such as bone grafting, guided tissue regeneration, or the use of biologics like enamel matrix derivative and platelet-rich fibrin are involved. The review also identifies critical knowledge gaps, including uncertainty regarding optimal treatment timing post-periodontal therapy, a lack of long-term and patient-centered outcomes, and the underrepresentation of adult-specific data in clinical research. Emerging technologies in tissue engineering, biomarker analysis, and digital orthodontic planning offer promising avenues for precision-based care. Ultimately, a collaborative, individualized approach that integrates biological insight with clinical expertise is key to achieving both periodontal stability and orthodontic success in periodontally vulnerable patients.

## Introduction

Orthodontic tooth movement (OTM) initiates a localized, aseptic inflammatory process in the periodontal ligament (PDL) and alveolar bone, driven by mechanical forces that lead to bone resorption on the pressure side and new bone formation on the tension side [1]. These forces alter blood flow and create hypoxic conditions in the PDL, triggering the release of inflammatory mediators and transient clinical signs such as redness, swelling, and pain responses essential for initiating tissue remodeling [2]. Periodontal cells, including PDL fibroblasts and osteocytes, act as mechanosensors. They respond to mechanical stress by secreting signaling molecules that recruit osteoclasts and osteoblasts to mediate bone resorption and deposition, respectively [2][3]. While the classical "compression-tension" theory serves as a foundational concept, the precise molecular mechanisms that translate mechanical stimuli into coordinated remodeling remain under investigation. This biomechanical-biological interface is central to effective and safe orthodontic practice [2].

Clinically, the interaction between orthodontics and the periodontium has become increasingly relevant, especially as more adults seek orthodontic treatment despite presenting with underlying periodontal conditions [3][4]. Active inflammation in periodontal tissues can exacerbate bone loss when subjected to orthodontic forces, particularly if hygiene is poor or periodontitis is unresolved [5].

However, when periodontal health is stabilized, orthodontic therapy may offer benefits such as improved occlusion, enhanced oral hygiene, and better esthetics, all of which contribute to long-term periodontal stability [6][7]. Biomechanical considerations such as using lighter forces and selecting appropriate appliances are essential for minimizing risks like root resorption or further tissue breakdown [5].

Beyond its clinical relevance, the orthodontic-periodontal interface offers a model for exploring mechanobiology and osteoimmunology. OTM integrates mechanical forces with immune responses, including systemic effects such as monocyte recruitment and cellular processes like autophagy in PDL cells [2][3]. Age-related changes in the periodontium, including heightened inflammation and reduced regenerative capacity, further complicate orthodontic treatment in adults, leading to slower tooth movement and greater susceptibility to adverse effects such as pain and root resorption [1][8] .

Given these complexities, there is a pressing need to re-examine the orthodontic-periodontal interface. This review evaluates current evidence on the biomechanical and biological mechanisms underlying periodontal remodeling in response to orthodontic forces. The aim is to provide a clinically relevant framework to optimize treatment outcomes while safeguarding periodontal health.

## Mechanobiology of Orthodontic Force and Periodontal Tissue Response

### Orthodontic Force Application and Tissue Stress-strain

**Figure-1:**
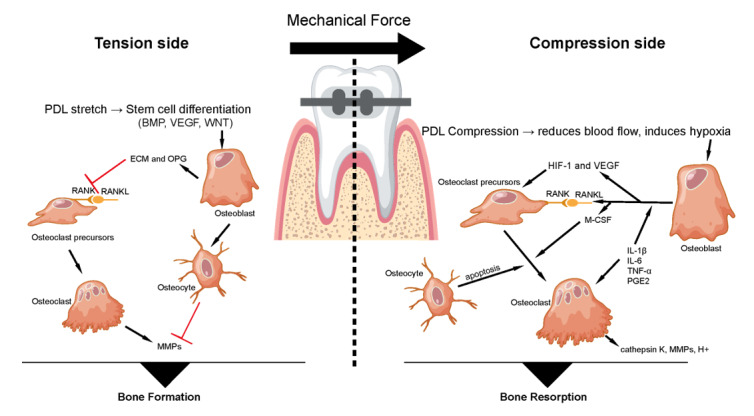
Figure 1. schematic illustrates orthodontic tooth movement under applied force, which generates a tension side and a compression side in the periodontal ligament and alveolar bone. On the tension side, osteoblasts (bone-forming cells) differentiate and secrete extracellular matrix proteins and osteoprotegerin (OPG), a decoy receptor that binds receptor activator of nuclear factor kappa-B ligand (RANKL), thereby inhibiting osteoclast activation. Osteocytes (mechanosensory cells embedded in bone) further support osteoblast function and bone deposition. On the compression side, osteoblasts release RANKL, which interacts with receptor activator of nuclear factor kappa-B (RANK) on osteoclast precursors, together with macrophage colony-stimulating factor (M-CSF), to promote their differentiation into mature osteoclasts. These multinucleated osteoclasts resorb bone through the secretion of matrix metalloproteinases (MMPs), cathepsin K, and hydrogen ions (H+). Additionally, osteocytes on the compression side may undergo apoptosis or secrete sclerostin, which inhibits osteoblast activity. The coordinated balance of bone formation on the tension side and bone resorption on the compression side enables controlled orthodontic tooth movement.

Orthodontic tooth movement (OTM) begins when mechanical force displaces a tooth within its socket, generating deformation (strain) in the surrounding periodontal ligament (PDL) and alveolar bone [9][10]. This initiates a well-characterized "compression-tension" response: PDL fibers compress on the pressure side and stretch on the tension side, accompanied by local changes in volume, fluid flow, and tissue architecture [3]. The extent of tissue strain is governed by both the force magnitude and the viscoelastic properties of the PDL, which displays nonlinear, time-dependent behavior [11] (Figure-1).

The PDL's biphasic composition solid fibers and interstitial fluid results in a two-phase mechanical response: an initial rapid displacement, followed by slower adjustment as fluid redistributes and the extracellular matrix adapts [11]. This dynamic stress-strain environment is key to initiating biological remodeling. Orthodontic forces also alter fluid movement in the bone’s lacuno-canalicular network: compression pushes fluid outward, while tension facilitates inward flow, generating shear stress that activates osteocytes, the primary mechanosensors in bone biology. Together, mechanical stress, matrix deformation, and fluid flow initiate the cascade of periodontal remodeling [3][11].

### PDL Biomechanics and Alveolar Bone Remodeling

The PDL is the first tissue to detect mechanical strain. Fibroblasts sense deformation through integrin-mediated adhesions that transmit force from the extracellular matrix to the cytoskeleton, activating intracellular signaling pathways such as MAPKs [11]. Within hours, these signals trigger the release of pro-inflammatory cytokines (e.g., ILs, prostaglandin E2) and growth factors that initiate sterile inflammation and recruit osteoclasts [11][12].

Alveolar bone remodeling is tightly coupled to PDL signaling [13]. On the pressure side, reduced perfusion induces PDL cells to release RANKL and other osteoclastogenic factors, promoting bone resorption [12][14]. On the tension side, cells express osteogenic mediators such as OPG and BMPs, promoting osteoblast differentiation and bone formation [14]. Osteocytes, embedded within the bone matrix, further modulate this response by detecting strain and shear stress, reinforcing the remodeling process [3].

One critical biomechanical consequence of excessive force is PDL hyalinization a localized zone of necrosis caused by extreme compression and vascular collapse [15]. This leads to a "lag phase" where tooth movement is temporarily halted until macrophages clear necrotic tissue and undermining resorption resumes [16]. These findings underscore the importance of applying forces within physiological limits to prevent treatment delays and tissue damage [15].

### Extracellular Matrix Remodeling in Orthodontic Tooth Movement

Orthodontic forces also drive remodeling of the PDL’s extracellular matrix (ECM), which is primarily composed of collagens (types I and III), fibronectin, laminin, and other glycoproteins [17]. Matrix turnover is regulated by matrix metalloproteinases (MMPs) and their inhibitors (TIMPs) [1][18]. On the pressure side, MMP activity increases to break down collagen and accommodate ligament compression, while the tension side shows upregulated TIMPs to inhibit MMPs and support new matrix deposition [1][19]. Specialized ECM proteins also contribute such as Periostin that is upregulated under high strain to stabilize collagen architecture [19]. Adhesion molecules such as fibronectin facilitate cell attachment, migration, and mechanotransduction [17]. After active orthodontic loading ceases, ECM remodeling continues during the retention phase, restoring tissue homeostasis [20].

### Variability of Tissue Response by Force Magnitude, Direction, and Duration

The biological response of periodontal tissues is strongly influenced by the mechanical parameters of applied force:

• Magnitude: Light, controlled forces stimulate efficient remodeling, while excessive forces cause hyalinization, root resorption, and bone loss [12]. The concept of an optimal orthodontic force (OOF) the smallest force that elicits near-maximal movement with minimal damage is well supported [12][21].

• Direction: Different types of tooth movement create distinct stress distributions. Intrusion concentrates forces apically and is associated with a high risk of root resorption [22], while bodily movement distributes stress along the root and is generally safer, albeit requiring higher force [23]. Clinicians manage these risks by adjusting vectors and mechanics [21][24].

• Duration: Force regimen matters. Continuous forces (e.g., fixed appliances) may overwhelm tissue recovery capacity, while intermittent forces (e.g., clear aligners or pause intervals) allow for vascular and cellular repair [25][26]. A 21-day-on/7-day-off regimen has been shown to reduce root resorption while preserving movement efficiency [25].

### Critical Insight: Key Mechanical Variables for Periodontal Tissue Preservation

Among all mechanical variables, force magnitude and force regimen are most critical. Applying the lowest biologically effective force preserves vascularity and cellular viability, favoring frontal resorption over undermining resorption [12]. Intermittent loading allows recovery and reduces adverse outcomes, especially in periodontally compromised or high-risk patients [25]. Controlling the direction of applied force ensures stress is distributed over broader PDL regions, reducing focal trauma. Altogether, these principles form the basis of biologically sensitive orthodontic therapy [11].

## Molecular and Cellular Remodeling Events

### Key Cellular Players in Orthodontic Remodeling

**Figure-2:**
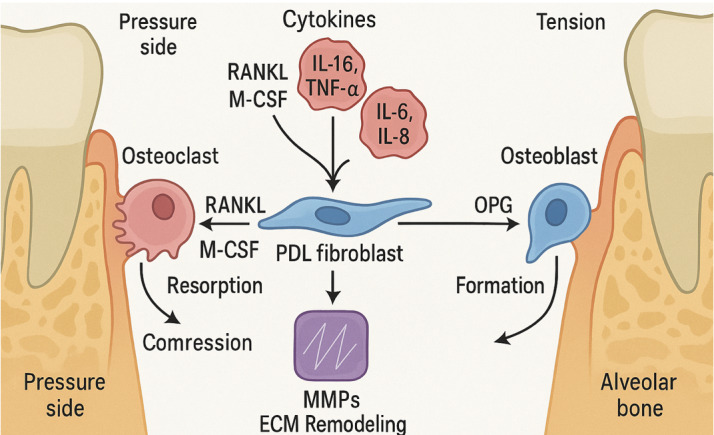
Figure 2. Key signaling pathways and cellular responses involved in periodontal tissue remodeling during orthodontic force application. On the pressure side (left), mechanical compression stimulates PDL fibroblasts to secrete RANKL and M-CSF, promoting osteoclast differentiation and bone resorption. These signals are amplified by early-release pro-inflammatory cytokines (IL-1β, IL-6, IL-8, TNF-α). On the tension side (right), PDL fibroblasts and osteoblasts secrete OPG, which inhibits RANKL activity and promotes new bone formation. Matrix metalloproteinases (MMPs), activated downstream of cytokine signaling, contribute to extracellular matrix (ECM) turnover and facilitate tissue adaptation.

Orthodontic tooth movement (OTM) is mediated by a complex network of cells that respond to mechanical stimuli with coordinated remodeling activities. Figure-2 illustrate the molecular and cellular remodeling events during orthodontic tooth movement.

Osteoclasts are the principal resorptive cells on the compression side. Derived from monocyte/macrophage precursors, their differentiation is stimulated by RANKL and M-CSF, which are secreted by PDL fibroblasts, osteoblasts, and osteocytes in response to compressive stress [27]. A rapid increase in the RANKL/OPG ratio within 24 hours reflects early osteoclastogenesis [14][27][28].

Osteoblasts, active on the tension side, arise from mesenchymal precursors and are responsible for new bone formation through deposition and mineralization of osteoid [29]. They also secrete OPG, which antagonizes RANKL, thereby regulating osteoclast activity and maintaining remodeling balance [30][31]. PDL fibroblasts serve as early mechanosensors. Under compression, they upregulate RANKL, M-CSF, PTHrP, and inflammatory mediators such as MCP-1 and prostaglandins, while downregulating OPG [32]. They also contribute to extracellular matrix (ECM) remodeling through synthesis of collagen and regulation of MMP/TIMP activity [33][34].

Endothelial cells respond to vascular compression by releasing VEGF, promoting angiogenesis and supporting immune cell infiltration [35]. TNF-α enhances VEGF signaling, which helps restore perfusion and deliver progenitor cells to sites of remodeling [36][37].

### Molecular Signaling Pathways in Remodeling

• RANKL/OPG Axis: This pathway is central to osteoclast regulation. RANKL binds to its receptor RANK on osteoclast precursors, promoting differentiation, while OPG acts as a decoy receptor, inhibiting this interaction [27]. Mechanical loading rapidly shifts the balance in favor of RANKL, particularly on the pressure side, leading to increased bone resorption [14][30]. Gingival crevicular fluid (GCF) studies confirm that this imbalance normalizes within 1-2 weeks, reflecting the resolution of the early inflammatory phase [27].

• Cytokine Cascade: Pro-inflammatory cytokines including IL-1β, IL-6, IL-8, and TNF-α are released early during OTM and promote osteoclastogenesis by enhancing RANKL expression and suppressing OPG [38][39]. IL-1β and TNF-α are particularly potent in resorptive signaling, and TNF-α also drives angiogenesis via VEGF induction [35]. Genetic polymorphisms in cytokine genes may influence individual variability in tooth movement rates and root resorption risk [40]. As remodeling progresses, anti-inflammatory cytokines (e.g., IL-4, IL-10) increase to resolve inflammation and support tissue repair [34][35].

• Matrix Metalloproteinases (MMPs): MMPs are key regulators of ECM remodeling. Collagenases (e.g., MMP-1, MMP-8) and gelatinases (e.g., MMP-2, MMP-9) break down collagen and denatured matrix components [34]. Their expression rises early after force application, peaks during active movement, and subsides as remodeling stabilizes [38][39]. MMP activity is balanced by tissue inhibitors of metalloproteinases (TIMPs), which modulate matrix turnover and maintain structural integrity [33][34].

### Temporal Dynamics of Orthodontic Remodeling

**Figure-3:**
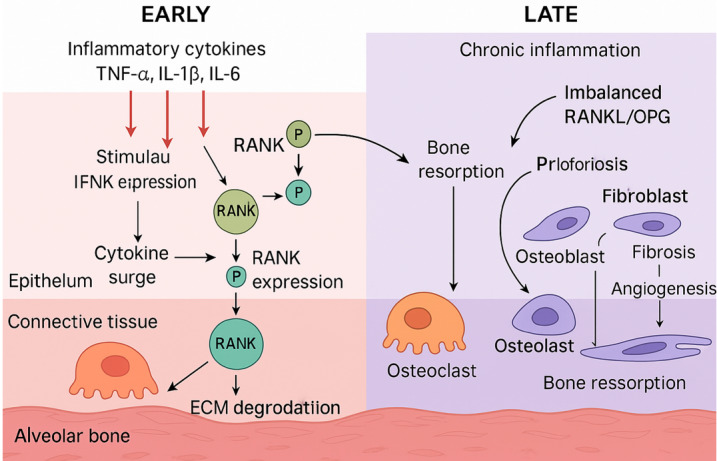
Figure 3. the dynamic molecular and cellular remodeling processes in periodontal tissue, divided into Early and Late phases. The Early phase depicts acute inflammation characterized by a surge of inflammatory cytokines (TNF-α, IL-1β, IL-6) leading to RANKL expression, activation of RANK on osteoclast precursors, ECM degradation via MMPs, and initial osteoclastogenesis. The Late phase illustrates chronic inflammation with an imbalanced RANKL/OPG axis promoting sustained osteoclast activity and bone resorption, accompanied by fibroblast proliferation, fibrosis, osteoblast recruitment, and angiogenesis. Cellular compartments such as epithelium, connective tissue, and alveolar bone are illustrated, with directional arrows indicating activation, and T-bars denoting inhibition or regulatory interactions. Phosphorylation events are marked with "P" symbols.

• Early Phase (Hours to Days): Characterized by vascular compression, ischemia, and an acute inflammatory response marked by increased cytokine and RANKL expression [27][34]. Osteoclast recruitment begins near marrow spaces, but due to necrosis and hyalinization, active movement is delayed a phenomenon known as the lag phase [1][27].

• Late Phase (Weeks Onward): Involves frontal resorption at the pressure side and osteogenesis at the tension side. Inflammatory mediators decline, the RANKL/OPG balance stabilizes, and sustained remodeling supports linear tooth movement with reduced discomfort [1][27][30]. Figure-3 represent the dynamic molecular and cellular remodeling processes in periodontal tissue, divided into Early and Late phases.

Understanding these temporal phases has clinical significance. For example, the use of NSAIDs may suppress early cytokine release and delay movement [27][41], while adjunctive therapies like low-level laser therapy or Piezocision aim to enhance early inflammation and accelerate remodeling [42].

## Impact of Periodontal Health Status

The periodontal status of adult patients plays a pivotal role in the safety and success of orthodontic treatment. Age-related changes in the periodontium such as reduced regenerative capacity, increased inflammation, and cumulative bone loss demand careful case selection and individualized treatment planning [4].

Periodontal disease, particularly in adults, often leads to complications such as pathological tooth migration, alveolar bone loss, and occlusal trauma [4][43]. In these patients, orthodontic forces applied without prior periodontal stabilization can accelerate tissue destruction, deepen pockets, and result in attachment loss [44]. Therefore, controlling inflammation and establishing periodontal stability are essential prerequisites before initiating tooth movement [45][46].

### Orthodontic Movement in Healthy vs. Compromised Periodontium

In patients with healthy periodontal tissues, orthodontic treatment is generally safe. Evidence shows that orthodontic forces produce minimal, often clinically negligible, attachment loss in healthy adults [43]. A meta-analysis reported an average loss of only ~0.1 mm in clinical attachment levels after treatment [45].

Even teeth with reduced but stable periodontal support can be moved predictably, provided there is no active inflammation [46].

Conversely, in a diseased periodontium, orthodontic forces can exacerbate destruction. Even mild gingival inflammation can lead to pronounced bone loss when coupled with mechanical loading [44][46].

In these cases, orthodontic intervention should be delayed until after completion of periodontal therapy and confirmation of inflammation resolution [46]. When performed appropriately, orthodontic treatment can support periodontal health by improving tooth alignment, reducing plaque retention sites, and distributing occlusal forces more evenly [43]. However, these benefits are only achievable with adequate plaque control and regular periodontal maintenance during treatment.

### Risk Factors for Adverse Periodontal Outcomes During Orthodontics

Multiple risk factors may increase susceptibility to tissue breakdown during orthodontic therapy:

Active periodontal inflammation: The most critical risk factor, which amplifies bone resorption under force [43][46].

Poor plaque control: Appliances complicate hygiene, increasing the risk of gingival inflammation and disease recurrence [4][47].

Excessive or poorly controlled forces: These can lead to root resorption, hyalinization, and rapid bone loss [43].

Pre-existing bone loss: Limits the safe envelope of tooth movement, especially for tipping or extrusion.

Thin gingival phenotype: Associated with a higher risk of dehiscence and gingival recession, particularly when teeth are moved outside the alveolar housing [48] .

Systemic factors: Smoking and poorly controlled diabetes reduce healing capacity and increase inflammation [4][43].

Despite these challenges, studies consistently show that, when appropriately timed and carefully managed, orthodontic treatment can be safely performed in periodontally compromised patients, often leading to functional and esthetic improvements [49][50].

### Timing and Sequencing of Periodontal Therapy

Proper timing is critical for successful outcomes. Orthodontic treatment should not begin until periodontal inflammation has been fully controlled [46]. Initial therapy including scaling, root planing, and oral hygiene instruction must precede appliance placement. Clinical stability should be confirmed through shallow probing depths and minimal bleeding on probing [51][52][53][54] .

In cases requiring regenerative surgery, traditional recommendations suggest a 3-6 month healing period before orthodontic forces are applied [55][56]. However, more recent studies support earlier intervention, often as soon as 4-8 weeks post-surgery, without compromising healing outcomes [46][56][57]. The decision should be individualized based on surgical complexity and patient response [46].

In selected cases, limited orthodontic movement (e.g., extrusion or alignment for debridement) may be justified during or shortly after periodontal therapy. However, full treatment should proceed only once long-term stability is evident [4] (Figure-3).

## Orthodontic–periodontal Regeneration Interplay with Grafts and Biologics

**Table:** Table1. Common regenerative materials used in orthodontic-periodontal therapy

**Material**	**Source**	**Advantages**	**Limitations**	**Orthodontic Implication**
**Autograft**	Patient’s own bone (intraoral or extraoral)	High osteogenic potential; no immune response	Donor site morbidity; limited quantity	Strong foundation for post-graft tooth movement
**Allograft**	Cadaveric human donor	Good biocompatibility; no donor site morbidity	Risk of disease transmission; variable quality	Commonly used prior to or during orthodontics
**Xenograft**	Animal-derived (typically bovine or porcine)	Readily available; good scaffold properties	Slower resorption; lower osteoinductivity	Effective in defect fill before alignment
**Synthetic Graft**	Synthetic materials (e.g., hydroxyapatite)	Customizable properties; avoids disease transmission	No osteoinductive properties; may integrate poorly	Useful for ridge preservation before movement
**Enamel Matrix Derivative (EMD)**	Porcine enamel protein extract	Promotes true periodontal regeneration (bone, cementum, PDL)	Technique sensitive; costlier than conventional grafts	Enhances tissue quality and attachment stability during/after movement
**Platelet-Rich Fibrin (PRF)**	Autologous blood-derived concentrate	Releases sustained growth factors; enhances healing	Short handling time; variable release profile	Supports soft and hard tissue healing post-surgery; safe for orthodontic application

### Integrating Regeneration into Orthodontic Planning

In patients with a history of periodontitis, the successful integration of orthodontic treatment often requires regenerative periodontal therapy. Alveolar bone defects such as vertical bone loss, dehiscence, or fenestration can limit safe tooth movement and increase the risk of further destruction if left untreated [58][59]. Moving teeth into uncorrected defects may worsen breakdown or even lead to tooth loss. As a result, regenerative techniques are frequently employed before or during orthodontic treatment to restore structural support and facilitate stable movement [60].

### Timing and Maintenance Around Regenerative Therapy

Clinical guidelines emphasize that all active periodontal disease must be controlled prior to orthodontic force application [58]. Once inflammation is resolved and regeneration has been performed, orthodontic forces may be initiated. Recent studies show that movement can safely begin as early as 4-8 weeks post-surgery, depending on healing status, without jeopardizing graft integrity or defect resolution [58][61]. Consistent periodontal maintenance, including professional cleanings every 2 to 6 months before, during, and after treatment, is essential to preserving regenerative outcomes and preventing disease recurrence [4][58].

### Regenerative Materials in Combined Therapy

Table-1 summarizing key regenerative materials used in orthodontic-periodontal therapy. A range of materials are used in combined orthodontic-periodontal protocols:

• Bone grafts:

o Autografts offer high osteogenic potential but involve donor site morbidity.

o Allografts and xenografts are commonly used alternatives; xenografts remodel more slowly [58][59].

o Synthetic substitutes (e.g., β-TCP, hydroxyapatite) provide an osteoconductive scaffold for bone fill and are useful in defect augmentation prior to movement.

• Barrier membranes in Guided Tissue Regeneration (GTR) are often paired with bone grafts. These membranes exclude epithelial cells from the defect, allowing selective repopulation by periodontal ligament and bone-forming cells. Clinical studies show that GTR improves attachment gain and defect fill, especially when followed by controlled tooth movement [60][62].

When orthodontic forces are applied to regenerated sites, light and controlled mechanics are essential. Successful movement through previously grafted areas has been demonstrated, provided healing is sufficient and forces are within physiological limits [60].

### Biologics Enhancing Periodontal Regeneration

Enamel Matrix Derivative (EMD) has emerged as one of the most studied biologics in periodontal regeneration. Applied during flap surgery, EMD stimulates cementogenesis, PDL regeneration, and bone formation [63]. Clinical protocols often combine EMD with autogenous or xenogeneic grafts for synergistic effects.

A systematic review showed that EMD use before orthodontic treatment improves the quality of regenerated tissue and attachment levels, particularly in complex defects [58].

In a study by Ogihara et al., orthodontic extrusion following EMD-enhanced regeneration led to significantly greater attachment gain in two-wall defects [64]. Long-term data from Tietmann et al. showed stable outcomes up to 10 years post-treatment when EMD was used in combination with orthodontic correction [65].

Platelet concentrates, such as platelet-rich plasma (PRP) and platelet-rich fibrin (PRF), are also gaining popularity. These biologics release key growth factors (PDGF, TGF-β, VEGF), accelerating healing and promoting soft and hard tissue regeneration [62][66]. PRF, in particular, offers sustained growth factor release due to its fibrin matrix and has been shown to outperform PRP in several contexts [66].

## Interdisciplinary Treatment Planning and Clinical Challenges

Effective management of patients requiring both orthodontic and periodontal treatment relies on close collaboration between orthodontists and periodontists. In cases of advanced periodontitis with secondary malocclusion, a multidisciplinary approach is essential to restore functional occlusion and esthetics while ensuring long-term periodontal stability [4]. Coordinated treatment planning demands early communication and clearly defined shared objectives so that both specialties can align interventions according to patient-specific needs [43]. Treatment goals must often be individualized and, at times, adjusted rather than pursuing ideal textbook outcomes, the team develops realistic targets that respect the patient’s reduced periodontal support, systemic factors, and compliance potential [4]. This pragmatic approach helps avoid overly ambitious orthodontic interventions that may compromise periodontal health or reduce patient adherence. Evidence supports this model: combined orthodontic-periodontal therapy has been shown to reduce relapse rates and achieve superior functional and esthetic outcomes compared to periodontal treatment alone [4].

### Case Selection and Treatment Staging

Comprehensive interdisciplinary planning begins with appropriate case selection and carefully staged treatment. Orthodontic movement should never be initiated in the presence of active periodontal disease (67). The periodontist must first resolve infections through initial therapy scaling, root planning, and risk factor control and confirm that disease parameters are stable (e.g., absence of probing depths ≥5 mm with bleeding) [4][43]. Initiating orthodontics during active inflammation risks accelerating attachment loss and compromising long-term outcomes [67]. Consequently, the initial stabilization phase may span 3 to 6 months or longer, depending on disease severity, during which the patient’s oral hygiene practices and periodontal response are closely monitored [67].

Only when the periodontist confirms that inflammation is resolved and the patient demonstrates consistent compliance should the orthodontist proceed with active tooth movement [39][67]. While some authors recommend waiting 3-6 months after non-surgical therapy or up to 12 months after regenerative surgery [4], emerging evidence challenges the necessity of such prolonged delays. A recent multicenter trial reported no significant differences in clinical outcomes whether orthodontic therapy began four weeks or six months post-surgery [4][56][64]. In well-maintained patients, early orthodontic intervention following periodontal therapy may yield similar gains in attachment and probing depth reduction compared to delayed approaches [4].

Current consensus guidelines, therefore, favor a case-by-case strategy: orthodontic treatment may begin once inflammation is controlled and the patient is enrolled in a maintenance program, rather than following arbitrary timelines [4]. The sequencing of specific procedures should also be individualized for example, regenerative bone grafting or soft tissue augmentation may be completed before orthodontics, while gingival contouring or implant placement is deferred until alignment is achieved. If signs of recurrent periodontal activity emerge during orthodontic care, active force application is paused until disease control is re-established [55][58][67].

### Patient Compliance and Maintenance

Patient selection must also include an assessment of compliance and hygiene capability, which are critical determinants of success in interdisciplinary cases. Orthodontic appliances increase plaque accumulation, complicating oral hygiene and elevating the importance of patient motivation and maintenance adherence [68]. Without consistent plaque control and frequent periodontal monitoring, even stable cases may relapse or deteriorate [4][47]. The interdisciplinary team should provide targeted hygiene instruction, reinforce preventive behaviors, and schedule regular maintenance visits with the periodontist throughout orthodontic care [43].

Evidence shows that patients who maintain excellent home care and comply with maintenance protocols exhibit significantly lower rates of recurrence and tooth loss compared to those who are non-compliant [4]. In contrast, orthodontic-periodontal therapy may be contraindicated or postponed in patients with poorly controlled diabetes, smoking habits, or inadequate hygiene [67]. Patient education is therefore a cornerstone of planning ensuring the individual understands that their cooperation is as critical as the treatment itself. Many clinicians implement a co-managed recall protocol (e.g., periodontal maintenance every 2-3 months during orthodontics) to monitor gingival health, probing depths, and signs of inflammation. Should relapse be detected, orthodontic force is suspended and periodontal re-treatment prioritized until stability is restored [67][68][69][70].

### Biomechanical Challenges: Root Resorption and Bone Loss

Orthodontic treatment in periodontally compromised patients presents unique biomechanical challenges [71]. Loss of alveolar bone alters the tooth’s center of resistance, increasing the lever arm and elevating the risk of uncontrolled tipping, root resorption, and unintended tooth movement [72]. Force systems must be customized accordingly light, biologically appropriate forces are preferred to minimize hyalinization and resorptive damage [73]. Cervical bracket positioning may help align force vectors with the new center of resistance, and segmented mechanics (rather than continuous arches) provide greater control in localized defect areas. Frequent radiographic monitoring and shorter activation intervals allow early detection of complications [67][74].

Despite precautions, mild root resorption is relatively common and can be exacerbated in sites with reduced bone support. However, even partial correction of pathologic migration or misalignment can improve periodontal load distribution and prognosis provided tooth movement stays within the biologic limits of the compromised periodontium [67]. Movements that risk pushing teeth beyond the alveolar housing (e.g., excessive labial translation in thin buccal plates) should be avoided. In such cases, adjunctive procedures such as alveolar augmentation or periodontally accelerated osteogenic orthodontics may be warranted to prevent dehiscence or recession [75].

### Addressing Attachment Loss and Esthetic Concerns

Orthodontic-periodontal cases often involve pre-existing attachment loss that presents esthetic challenges during and after treatment. One common issue is the development of "black triangles" (open gingival embrasures) following alignment of teeth that have lost interproximal bone and papilla. These spaces are both cosmetic and functional concerns, promoting food impaction and complicating hygiene [76][77].

To mitigate black triangles, orthodontists may employ minor intrusion or interproximal reduction to reduce the distance between contact points and the bone crest, promoting partial papilla fill [78]. However, full soft tissue regeneration is unpredictable, and in severe cases adjunctive esthetic procedures are required [79]. Periodontists may perform papilla augmentation or connective tissue grafts, while restorative dentists can use bonding or veneers to reshape teeth and close spaces [67].

Another concern is gingival recession on prominent roots, especially in proclined incisors. Orthodontic movement cannot reverse recession and, if poorly planned, may worsen it. While controlled movement in a healthy periodontal environment does not appear to increase attachment loss significantly [43], labial expansion and extrusion in thin biotypes are known risk factors for additional recession [4].

In such cases, preventive soft tissue grafting (e.g., free gingival or connective tissue grafts) prior to orthodontics may be considered [75]. Most root coverage procedures are deferred until post-treatment, when tooth positions are ideal for grafting or restorative care [67]. In scenarios requiring expansion or complex movement, combined surgical-orthodontic approaches such as corticotomies with grafting may be employed to generate new bone and protect against fenestration or recession [4].

Through proactive management of these esthetic and structural challenges, the interdisciplinary team can deliver not only a functional result but one that meets the esthetic expectations of adult patients [75].

## Future Directions and Knowledge Gaps

Despite significant progress in understanding orthodontic-periodontal interactions, several controversies and unresolved questions remain. A central debate concerns whether orthodontic treatment can meaningfully enhance periodontal outcomes in compromised patients [72].

Current evidence suggests only marginal improvements typically a few tenths of a millimeter in clinical attachment level or bone height when orthodontic treatment is combined with periodontal therapy [43]. To date, no robust controlled trials have demonstrated that orthodontic tooth movement independently improves or worsens long-term periodontal health, underscoring a fundamental gap in the evidence base [80].

The long-term stability of orthodontically induced periodontal regeneration also remains uncertain. Although initial gains in bone fill or attachment may be observed, these results can be inconsistent, and relapse is a persistent concern. Identifying factors that sustain periodontal improvements after treatment is therefore a critical research priority [50][65][80].

Another ongoing area of uncertainty involves the optimal timing of orthodontic intervention following periodontal therapy. Existing guidelines are primarily based on clinical experience rather than high-quality evidence, reflecting a lack of consensus regarding the safest and most effective window for initiating tooth movement after periodontal healing [4][56]. Additionally, adult patients tend to exhibit slower tooth movement, a heightened inflammatory response, and increased susceptibility to complications such as root resorption and orthodontic pain compared to adolescents [1]. However, many studies do not stratify results by age, leaving these age-related differences in periodontal remodeling underexplored and unaddressed. This represents a notable gap in optimizing orthodontic protocols for adult patients [81].

Methodological limitations further constrain the current body of research on orthodontic-periodontal therapy [82]. Many studies are limited by small sample sizes, heterogeneous designs, short follow-up durations, and inconsistent outcome measures, which complicate comparisons and reduce the generalizability of findings [43].

Publication and reporting biases, including the preferential publication of positive outcomes, may further skew the evidence, making it difficult to assess true effect sizes [4]. In particular, patient-centered outcomes are frequently neglected. Quality of life metrics and patient satisfaction despite being central to treatment goals are rarely included in study designs [67]. The limited attention to patient-reported outcomes (PROs) constitutes a significant gap in understanding the full impact of these interventions on daily life and long-term well-being [83].

To address these limitations, future studies must adopt more rigorous and transparent research methodologies [84].

Multicenter randomized controlled trials with standardized treatment protocols, extended follow-up periods, and clearly defined clinical and patient-reported outcome measures are essential for producing high-quality, reliable evidence [80]. Adequate sample sizes and age stratification should be prioritized to ensure subgroup-specific insights. Furthermore, transparent reporting including the publication of negative or inconclusive results is necessary to reduce bias and guide evidence-based clinical practice [80][84].

Looking ahead, researchers are developing innovative models and technologies to deepen the understanding of orthodontic and periodontal tissue remodeling. Future investigations are expected to examine the long-term effects of specific orthodontic techniques such as intrusion of extruded teeth or molar up righting on periodontal outcomes in adult patients [4]. In parallel, advances in regenerative medicine and tissue engineering offer exciting prospects. The integration of stem cells, growth factors, and bioactive scaffolds into orthodontic care may help facilitate new periodontal tissue formation during tooth movement, enhancing both healing and long-term stability [4]. Technological innovations are also poised to play a transformative role. Customized biomaterials, including bioactive orthodontic adhesives and antimicrobial coatings, are being developed to reduce plaque accumulation and improve post-treatment stability [4][85].

The increasing use of clear aligners in periodontally susceptible patients is another area of interest, as aligners may facilitate better oral hygiene and reduce inflammation compared to fixed appliances [85]. In addition, predictive tools such as computational models that incorporate patient-specific data (e.g., bone density, biomarker profiles in gingival crevicular fluid) are being explored to personalize force application and minimize adverse outcomes [86].

These biological and technological innovations, when combined with high-quality research designs, are expected to generate more effective and patient-centered orthodontic strategies that support periodontal health [85][86]. Moreover, there is growing consensus that interdisciplinary collaboration is essential for optimizing outcomes in periodontally vulnerable adults. Orthodontic treatment in these patients should be coordinated closely with periodontists and other specialists to ensure comprehensive care planning that addresses both alignment and periodontal stability [80]. A multidisciplinary approach enables individualized consideration of each patient’s periodontal condition, systemic risk factors, and aesthetic goals, thereby improving long-term treatment success and reducing complications [80][86].

Future research should also integrate real-world data and patient-reported outcome measures to ensure that clinical advances align with patient priorities. By focusing on outcomes that matter most to patients such as comfort, satisfaction, and functional improvement research can inform clinical protocols that truly enhance quality of life [80][85].

## Conclusion

The interaction between orthodontics and periodontal tissue remodeling is inherently multifactorial, reflecting a dynamic interplay of mechanical forces, cellular activity, molecular signaling, and clinical context. Orthodontic tooth movement is mediated by tightly regulated processes involving osteoclasts, osteoblasts, fibroblasts, and endothelial cells, orchestrated through pathways such as RANKL/OPG balance, cytokine cascades, and matrix metalloproteinase activity. These biological responses are further modulated by patient-specific factors, including baseline periodontal health, systemic status, and treatment compliance.

Across clinical scenarios, evidence underscores that orthodontic outcomes cannot be divorced from the periodontal environment. In periodontally healthy adults, tissue remodeling generally follows predictable biological phases, while in compromised tissues, risks of bone loss, attachment reduction, and esthetic concerns require careful staging and adjunctive periodontal therapy.

Advances in regenerative materials, scaffold-based strategies, and bioengineering hold promise for restoring lost periodontal support and enhancing stability when integrated with orthodontic mechanics.

Emerging technologies ranging from salivary and gingival biomarkers to multi-omics approaches, AI-driven predictive models, and 3D imaging of soft tissues are beginning to transform our capacity to monitor and anticipate periodontal responses. However, current evidence is limited by small-scale studies, heterogeneity in methodology, and a lack of long-term outcomes, underscoring the need for multicenter, interdisciplinary trials with standardized protocols.

Taken together, the future of orthodontic-periodontal care lies in integrated, precision-oriented strategies that align mechanical, biological, and technological insights. Collaborative treatment planning between orthodontists and periodontists is essential not only to reduce complications but also to optimize functional, structural, and esthetic outcomes. As research progresses, translating emerging biological and digital tools into personalized care pathways will be critical to improving stability, patient-centered outcomes, and long-term oral health.

## Conflict of Interest

None.

## References

[R1] Wang J, Huang Y, Chen F, Li W (2024). The agerelated effects on orthodontic tooth movement and the
surrounding periodontal environment. Front Physiol.

[R2] Li B, Wang L, He H (2025). Autophagy in orthodontic tooth movement: advances, challenges,
and future perspectives. Mol Med.

[R3] Li Y, Zhan Q, Bao M, Yi J, Li Y (2021). Biomechanical and biological responses of periodontium in
orthodontic tooth movement: update in a new decade. Int J Oral Sci.

[R4] Zhong W, Zhou C, Yin Y, Feng G, Zhao Z, Pan Y, et al (2025). Expert consensus on orthodontic treatment of patients with
periodontal disease. Int J Oral Sci.

[R5] Viglianisi G, Polizzi A, Lombardi T, Amato M, Grippaudo C, Isola G (2025). Biomechanical and Biological Multidisciplinary Strategies in the
Orthodontic Treatment of Patients with Periodontal Diseases: A Review of the
Literature. Bioengineering.

[R6] Oruba Z, Gibas-Stanek M, Pihut M, Cześnikiewicz-Guzik M, Stós W (2023). Orthodontic treatment in patients with periodontitis – a
narrative literature review. Aust Dent J.

[R7] Antonarakis GS, Zekeridou A, Kiliaridis S, Giannopoulou C (2024). Periodontal considerations during orthodontic intrusion and
extrusion in healthy and reduced periodontium. Periodontol 2000.

[R8] Saad NA, Ariffin F (2025). Orthodontic and periodontal health interplay: insight from a case
series. IIUM J Orofac Health Sci.

[R9] Feştilă D, Ciobotaru CD, Condurache P, Kahane L, Şimon CI, Chiş A, et al (2024). Maintenance of periodontal health during adult orthodontic
treatment. Med Evol.

[R10] Alghamdi B, Jeon HH, Ni J, Qiu D, Liu A, Hong JJ, et al (2023). Osteoimmunology in Periodontitis and Orthodontic Tooth Movement. Curr Osteoporos Rep.

[R11] Maltha JC, KuijpersJagtman AM (2023). Mechanobiology of orthodontic tooth movement: An update. J World Fed Orthod.

[R12] Zheng W, Lu X, Chen G, Shen Y, Huang X, Peng J, et al (2024). The osteoclastic activity in apical distal region of molar mesial
roots affects orthodontic tooth movement and root resorption in rats. Int J Oral Sci.

[R13] Nakai Y, Praneetpong N, Ono W, Ono N (2023). Mechanisms of Osteoclastogenesis in Orthodontic Tooth Movement
and Orthodontically Induced Tooth Root Resorption. J Bone Metab.

[R14] Yang CY, Jeon HH, Alshabab A, Lee YJ, Chung CH, Graves DT (2018). RANKL deletion in periodontal ligament and bone lining cells
blocks orthodontic tooth movement. Int J Oral Sci.

[R15] Jamali S, Khosravi S, Shadmanpour M, Gharibpour F, Payahoo S, Darvish M (2020). Hyalinization and Molecular Pathways Involved in Orthodontic
Tooth Movement: A Systematic Review and MetaAnalysis. Pesqui Bras Em Odontopediatria E Clínica Integrada.

[R16] Chaushu S, Klein Y, Mandelboim O, Barenholz Y, Fleissig O (2022). Immune Changes Induced by Orthodontic Forces: A Critical Review. J Dent Res.

[R17] Yu QY, Huang YP, Li WR (2024). Extracellular Matrix Remodelling of the Periodontium under
Orthodontic Force. Chin J Dent Res.

[R18] Behm C, Nemec M, Weissinger F, Rausch MA, Andrukhov O, Jonke E (2021). MMPs and TIMPs Expression Levels in the Periodontal Ligament
during Orthodontic Tooth Movement: A Systematic Review of In Vitro and In
Vivo Studies. Int J Mol Sci.

[R19] Chiu KH, Karpat M, Hahn J, Chang KY, Weber M, Wolf M, et al (2023). Cyclic Stretching Triggers Cell Orientation and Extracellular
Matrix Remodeling in a Periodontal Ligament 3D In Vitro Model. Adv Healthc Mater.

[R20] Vansant L, Cadenas De, Verdonck A, Willems G (2018). Expression of biological mediators during orthodontic tooth
movement: A systematic review. Arch Oral Biol.

[R21] Zhu Z, Sun X, Lu B, Shi Q, Tang Y, Zou S, et al (2025). The Finite Element Analysis of Optimal Orthodontic Force for
Canine Distalization with LongArm Brackets. J Biosci Med.

[R22] Akl HE, ElBeialy AR, ElGhafour MA, Abouelezz AM, El Sharaby (2021). Root resorption associated with maxillary buccal segment
intrusion using variable force magnitudes. Angle Orthod.

[R23] Gupta S, Gupta G, Sharma M, Sharma PA, Goyal S, Kumar P (2015). An Evaluation of the Stress Pattern Distribution for Orthodontic
Tooth Movements – A Finite Element Study. Dent J Adv Stud.

[R24] Moga R, Olteanu C, Delean A (2024). Ischemic Risks Induced by Larger Orthodontic Forces on Dental
Pulp and NeuroVascular Bundle in Reduced Periodontium. J Clin Med.

[R25] Ghaleb S, Tamish N, ElKenany W, Guindi M (2021). The effect of two different types of forces on possible root
resorption in relation to dentin phosphoprotein levels: a singleblind,
splitmouth, randomized controlled trial. Prog Orthod.

[R26] Ozkalayci N, Karadeniz E, ElekdagTurk S, Turk T, Cheng LL, Darendeliler M (2018). Effect of continuous versus intermittent orthodontic forces on
root resorption: A microcomputed tomography study. Angle Orthod.

[R27] Danz JC, Degen M (2025 Mar 6 [cited 2025 Aug 25]). Selective modulation of the bone remodeling regulatory system through
orthodontic tooth moveme.

[R28] Ono T, Nakashima T (2018). Recent advances in osteoclast biology. Histochem Cell Biol.

[R29] Udagawa N, Takahashi N, Jimi E, Matsuzaki K, Tsurukai T, Itoh K, et al (1999). Osteoblasts/stromal cells stimulate osteoclast activation through
expression of osteoclast differentiation factor/RANKL but not macrophage
colonystimulating factor. Bone.

[R30] Kumar DrIG, Raghunath DrN, H DrJ (2022). RANKRANKLOPG: A current trends in orthodontic tooth movement and
its role in accelerated orthodontics. Int J Appl Dent Sci.

[R31] Glass DA, Bialek P, Ahn JD, Starbuck M, Patel MS, Clevers H, et al (2005). Canonical Wnt Signaling in Differentiated Osteoblasts Controls
Osteoclast Differentiation. Dev Cell.

[R32] Asano M, Yamaguchi M, Nakajima R, Fujita S, Utsunomiya T, Yamamoto H, et al (2011). IL-8 and MCP-1 induced by excessive orthodontic force mediates
odontoclastogenesis in periodontal tissues. Oral Dis.

[R33] Lin T, Yang L, Zheng W, Zhang B (2021). Matrix metalloproteinases and Th17 cytokines in the gingival
crevicular fluid during orthodontic tooth movement. Eur J Paediatr Dent.

[R34] Inchingolo F, Inchingolo AM, Malcangi G, Ferrante L, Trilli I, Di Noia, et al (2024). The Interaction of Cytokines in Orthodontics: A Systematic
Review. Appl Sci.

[R35] Noguchi T, Kitaura H, Marahleh A, Ohori F, Nara Y, Pramusita A, et al (2022). Tumor necrosis factorα enhances the expression of vascular
endothelial growth factor in a mouse orthodontic tooth movement model. J Dent Sci.

[R36] Nguyen VT, Nardini M, Ruggiu A, Cancedda R, Descalzi F, Mastrogiacomo M (2020). Platelet Lysate Induces in Human Osteoblasts Resumption of Cell
Proliferation and Activation of Pathways Relevant for Revascularization and
Regeneration of Damaged Bone. Int J Mol Sci.

[R37] Zhang L, Wang M, Qiu H, Wei Y, Zhou L, Nian N, et al (2023). Epicatechin gallate promotes vascularization in coculture of
human osteoblasts and outgrowth endothelial cells. Exp Biol Med.

[R38] Garlet TP, Coelho U, Silva J, Garlet G (2007). Cytokine expression pattern in compression and tension sides of
the periodontal ligament during orthodontic tooth movement in humans. Eur J Oral Sci.

[R39] AvornicCiumeico L, Trifan V, Uzun I, Calfa S, Zumbreanu I, Ciumeico I (2024 June [cited 2025 Aug 25]). The role of the inflammatory process in orthodontic too.

[R40] Behnaz M, Jazaeri M, Aghandeh P, Taheri M, GhafouriFard S (2020). Genetic factors in determination of risk of external apical root
resorption: A concise review. Gene Rep.

[R41] Yamaguchi M, Fukasawa S (2021). Is Inflammation a Friend or Foe for Orthodontic Treatment.:
Inflammation in Orthodontically Induced Inflammatory Root Resorption and
Accelerating Tooth Movement. Int J Mol Sci.

[R42] CastilloMontaño L, ColinoGallardo P, BaptistaSanchez H, Drewling I, AlvaradoLorenzo M, AntonioZancajo L, et al (2024). Efficacy of Invasive and NonInvasive Methods in Orthodontic Tooth
Movement Acceleration: A Systematic Review. Appl Sci.

[R43] Fleming PS, Andrews J (2024). Periodontitis: orthodontic implications and management. Br Dent J.

[R44] Ristoska S, Dzipunova B, Stefanovska E, Rendzova V, RadojkovaNikolovska V, Evrosimovska B (2019). Orthodontic Treatment of a Periodontally Affected Adult Patient
(Case Report). Open Access Maced J Med Sci.

[R45] Papageorgiou SN, Papadelli AA, Eliades T (2018). Effect of orthodontic treatment on periodontal clinical
attachment: a systematic review and metaanalysis. Eur J Orthod.

[R46] Jepsen K, Sculean A, Jepsen S (2023). Complications and treatment errors involving periodontal tissues
related to orthodontic therapy. Periodontol 2000.

[R47] Peterson BW, Tjakkes G, Renkema A, Manton DJ, Ren Y (2024). The oral microbiota and periodontal health in orthodontic
patients. Periodontol 2000.

[R48] Zargham A, Geramy A, Rouhi G (2016). Evaluation of longterm orthodontic tooth movement considering
bone remodeling process and in the presence of alveolar bone loss using
finite element method. Orthod Waves.

[R49] Almutairi RM, Alturaif DJ, Alanzi LM (2023). Importance of Oral Hygiene in Orthodontic Treatment. Saudi J Oral Dent Res.

[R50] Shumynska T, Melnichuk T (2025). Longterm results of treatment of periodontal diseases in children
in the dynamics of orthodontic treatment with fixed appliances. Suchasna Stomatol.

[R51] Akbar A, Chheena A, Mansoor S, Zulfiqar RMF, Saeed F, Butt H (2024). Effect of Periodontal Scaling in Patients Undergoing Orthodontic
Treatment on General Oral and Periodontal Health. J Health Rehabil Res.

[R52] Erbe C, Heger S, Kasaj A, Berres M, Wehrbein H (2022). Orthodontic treatment in periodontally compromised patients: a
systematic review. Clin Oral Investig.

[R53] Gehlot M, Sharma R, Tewari S, Kumar D, Gupta A (2022). Effect of orthodontic treatment on periodontal health of
periodontally compromised patients. Angle Orthod.

[R54] Krausz E, Einy S, Aizenbud D, Levin L (2011). Orthodontic treatment in periodontal patients. Refuat HaPeh VehaShinayim.

[R55] Tietmann C, Bröseler F, Axelrad T, Jepsen K, Jepsen S (2021). Regenerative periodontal surgery and orthodontic tooth movement
in stage IV periodontitis: A retrospective practice-based cohort study. J Clin Periodontol.

[R56] Jepsen K, Tietmann C, Kutschera E, Wüllenweber P, Jäger A, Cardaropoli D, et al (2021). The effect of timing of orthodontic therapy on the outcomes of
regenerative periodontal surgery in patients with stage IV periodontitis: A
multicenter randomized trial. J Clin Periodontol.

[R57] Tu CC, Lo CY, Chang PC, Yin HJ (2022). Orthodontic treatment of periodontally compromised teeth after
periodontal regeneration: A restrospective study. J Formos Med Assoc.

[R58] Deandra FA, Sulijaya B, Sudjatmika DA, Harsas NA (2024). Selection of bone graft material and proper timing of periodontal
surgery for orthodontic patients: A systematic review. Heliyon.

[R59] Miao Y, Chang YC, Tanna N, Almer N, Chung CH, Zou M, et al (2022). Impact of Frontier Development of Alveolar Bone Grafting on
Orthodontic Tooth Movement. Front Bioeng Biotechnol.

[R60] Martin C, Sanz M (2024). Orthodontic tooth movement after periodontal regeneration of
intrabony defects. Korean J Orthod.

[R61] Ghezzi C, Viganò VM, Francinetti P, Zanotti G, Masiero S (2013). Orthodontic Treatment After Induced Periodontal Regeneration in
Deep Infrabony Defects. Clin Adv Periodontics.

[R62] Ashfaq R, Kovács A, Berkó S, BudaiSzűcs M (2024). Developments in Alloplastic Bone Grafts and Barrier Membrane
Biomaterials for Periodontal Guided Tissue and Bone Regeneration Therapy. Int J Mol Sci.

[R63] Xiang C, Zhang L, Tao E (2025). Research progress of enamel matrix derivative on periodontal
tissue regeneration: a narrative review. Front Dent Med.

[R64] Ogihara S, Wang H (2010). Periodontal Regeneration With or Without Limited Orthodontics for
the Treatment of 2- or 3-Wall Infrabony Defects. J Periodontol.

[R65] Tietmann C, Jepsen S, Heibrok H, Wenzel S, Jepsen K (2023). Long-term stability of regenerative periodontal surgery and
orthodontic tooth movement in stage IV periodontitis: 10-year data of a
retrospective study. J Periodontol.

[R66] Ardila CM, Pertuz M, VivaresBuiles AM (2023). Clinical Efficacy of Platelet Derivatives in Periodontal Tissue
Regeneration: An Umbrella Review Casarin R, editor. Int J Dent.

[R67] Feu D (2020). Orthodontic treatment of periodontal patients: challenges and
solutions, from planning to retention. Dent Press J Orthod.

[R68] Isler AAA (2022). Orthodontıc treatment and oral flora. Asian J Med Biol Res.

[R69] Roberts HM, Yonel Z, Kantarci A, Grant MM, Chapple IL (2022). Impact of gingivitis on circulating neutrophil reactivity and
gingival crevicular fluid inflammatory proteins. Int J Environ Res Public Health.

[R70] Chatzisymeonidou S, Papadopoulos P, Andreadis D, Poulopoulos A (2023). Desquamative gingivitis: Clinical and epidemiological findings in
patients from Northern Greece. Balk J Dent Med.

[R71] Sum FHKMH, Shan Z, Chan YHD, Chu RJDH, Pelekos G, She TT (2024). Biomechanical Considerations in the Orthodontic Treatment of a
Patient with Stabilised Stage IV Grade C Generalised Periodontitis: A Case
Report. Bioengineering.

[R72] Zhang Y, Yan J, Zhang Y, Liu H, Han B, Li W (2024). Agerelated alveolar bone maladaptation in adult orthodontics:
finding new ways out. Int J Oral Sci.

[R73] Ugarte OM, Cattaneo PM, Roscoe MG, Onone IG, Dominguez GC, Meira JBC (2022). Optimal Intrusive Force for a Periodontally Compromised Tooth: A
Finite Element Analysis Strategy. Dent Mater.

[R74] Sabbagh H, Haas E, Baumert U, Seidel CL, Hötzel L, Wichelhaus A (2024). Biomechanical Simulation of Orthodontic EnBloc Retraction
Comparing Compound Technique and Sliding Mechanics Using a HOSEA Robotic
Device. Bioengineering.

[R75] Kalina E, Machoy M, Górski B (2024). Interdisciplinary Approaches by Polish Orthodontists,
Periodontists, and Oral Surgeons to Soft Tissue Augmentation in Adult
Patients: A Survey Study. Appl Sci.

[R76] Rashid ZJ, Gul SS, Shaikh MS, Abdulkareem AA, Zafar MS (2022). Incidence of Gingival Black Triangles following Treatment with
Fixed Orthodontic Appliance: A Systematic Review. Healthcare.

[R77] Jung JS, Lim HK, Lee YS, Jung SK (2024). The Occurrence and Risk Factors of Black Triangles Between
Central Incisors After Orthodontic Treatment. Diagnostics.

[R78] Patel M, Guni A, Nibali L, GarciaSanchez R (2024). Interdental papilla reconstruction: a systematic review. Clin Oral Investig.

[R79] Dong J, Liao Y, Sun M, Gong Y, Chen H, Song Z (2023). Modified interproximal tunneling technique with customized
subepithelial connective tissue graft for gingival papilla reconstruction:
report of three cases with a cutback incision on the palatal side. BMC Oral Health.

[R80] Hashim NT, Dasnadi SP, Ziada H, Rahman MM, Ahmed A, Mohammed R, et al (2025). Orthodontic Management of Different Stages and Grades of
Periodontitis According to the 2017 Classification of Periodontal Diseases. Dent J.

[R81] Yan T, Li H, Yan J, Ma S, Tan J (2024). Age-related mitophagy regulates orthodontic tooth movement by
affecting PDLSCs mitochondrial function and RANKL/OPG. FASEB J.

[R82] Kim H, Jo H, Cha JY, Lee KJ, Yu HS, Choi SH (2024). Orthodontic treatment of a middleaged patient with periodontally
compromised dentition accompanied by pathologic tooth migration. Angle Orthod.

[R83] Salvesen BF, Grytten J, Rongen G, VandevskaRadunovic V (2022). PatientReported Outcome Measures on Oral Hygiene, Periodontal
Health, and Treatment Satisfaction of Orthodontic Retention Patients up to
Ten Years after Treatment A CrossSectional Study. Int J Environ Res Public Health.

[R84] Almasri AMH, Hajeer MY, Ajaj MA, Almusawi AOA, Jaber ST, Zakaria AS, et al (2024 July 25 [cited 2025 Sept 4]). Patient Satisfaction Following Orthodontic Treatment A System.

[R85] Dipalma G, Inchingolo AD, Fiore A, Balestriere L, Nardelli P, Casamassima L, et al (2025). The Differential Impact of Clear Aligners and Fixed Orthodontic
Appliances on Periodontal Health: A Systematic Review. Children.

[R86] Zhao J, Feng Z, Liu Y, Sun S, Feng Z (2025). Advances in orthodontic treatment for periodontal disease: a
bibliometric analysis, emerging insights and clinical implications. Front Dent Med.

